# Evaluating the utility of a two‐assay serological algorithm for hepatitis C screening in a low prevalence population

**DOI:** 10.1002/jcla.24887

**Published:** 2023-04-27

**Authors:** Kai J. Rogers, Tracy S. Halvorson, Matthew D. Krasowski, Anna E. Merrill

**Affiliations:** ^1^ Department of Pathology University of Iowa Hospitals and Clinics Iowa City Iowa USA

**Keywords:** hepatitis C, prevalence, serology

## Abstract

**Introduction:**

Screening for hepatitis C virus (HCV) is performed by testing for anti‐HCV antibodies, which may yield false‐positive results leading to additional testing and other downstream consequences for the patient. We report our experience in a low prevalence population (<0.05%) using a two‐assay algorithm aimed at testing specimens with borderline or weak positive anti‐HCV reactivity in the screening assay by a second anti‐HCV assay prior to confirming positive anti‐HCV results with RT‐PCR.

**Materials and Methods:**

Retrospective analysis of 58,908 plasma samples was obtained over a 5‐year period. Samples were initially tested using the Elecsys Anti‐HCV II assay (Roche Diagnostics), with borderline or weakly positive results (defined in our algorithm as a Roche cutoff index of 0.9–19.99) reflexively analyzed using the Architect Anti‐HCV assay (Abbott Diagnostics). The Abbott anti‐HCV results dictated the final anti‐HCV interpretation for reflexed samples.

**Results:**

Our testing algorithm resulted in 180 samples requiring second‐line testing, with final anti‐HCV results interpreted as 9% positive, 87% negative, and 4% indeterminate. The positive predictive value (PPV) of a weakly positive Roche result was 12%, which was significantly lower than the PPV using our two‐assay approach (65%).

**Conclusions:**

The incorporation of a two‐assay serological testing algorithm in a low prevalence population provides a cost‐effective method of improving the PPV of HCV screening in specimens with borderline or weakly positive anti‐HCV results.

## Graphical abstract

Screening for hepatitis C virus antibodies may yield false positive results leading to additional testing and other downstream consequences for the patient. We report our experience in a low prevalence population (<0.05%) using a two‐assay algorithm aimed at testing specimens with borderline or weak positive anti‐HCV reactivity in the screening assay by a second anti‐HCV assay prior to confirming positive anti‐HCV results with RT‐PCR. This approach increases the positive predictive value from 12% to 65%, providing a cost‐effective method of HCV screening in specimens with borderline or weakly positive anti‐HCV results.

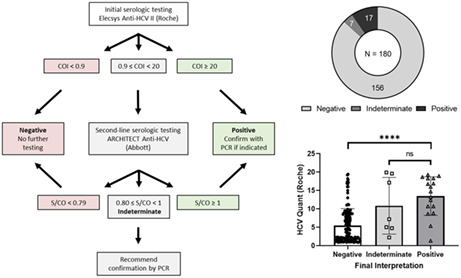

## 1 INTRODUCTION

Hepatitis C virus (HCV), a positive strand RNA virus and member of the family *Flaviviridae*, is a bloodborne pathogen that was historically associated with blood transfusion but is now primarily spread via shared needles or other equipment used to prepare and inject drugs. While HCV can cause severe acute illness, many cases are not detected in the early stages. As a result, most HCV‐associated morbidity and mortality arises from long‐term complications of chronic infection including liver cirrhosis, hepatocellular carcinoma, and decompensated liver failure.^1^, ^2^ With the advent of novel therapies, infection with HCV has become curable in those able to access and afford treatment, and the prevalence of chronic HCV has slowly, yet consistently, declined. Unfortunately, given the financial barriers associated with these treatments, these therapies are not uniformly available, further illuminating the health disparities that plague even the most resource‐rich countries. In contrast to the trends observed with chronic HCV, acute HCV infections have increased dramatically in the United States in the last 15 years, driven in large part by the ongoing opioid epidemic and associated intravenous drug use.^3^ While trends regarding the incidence of acute HCV vary in different parts of the world, largely mirroring trends in intravenous drug use, it now predominantly occurs in younger adults, disproportionately impacting vulnerable communities that may lack access to high quality, consistent health care. These epidemiologic shifts highlight the importance of successful screening programs in identifying patients infected with HCV and facilitating early access to care.^4^

Per guidelines from the United States Centers for Disease Control and Prevention (CDC), screening for HCV is based on the detection of circulating anti‐HCV antibodies and should be performed once for all adults over age 18, for pregnant women in each pregnancy, and routinely in individuals at high risk of infection. Routine, repeated HCV testing is also performed in specific clinical settings such as for patients undergoing hemodialysis.^5^ The most common screening approach in the United States is testing with a single anti‐HCV assay, with subsequent confirmation of positive results by detection of HCV RNA. One limitation to this approach is false‐positive anti‐HCV results, which are defined as a positive screening result in the absence of additional laboratory or clinical evidence of HCV infection. These false positives can lead to myriad adverse downstream consequences including erroneous diagnoses, additional testing, and unnecessary alerting of public health officials. Although commercially available anti‐HCV assays demonstrate high specificity, widespread screening can result in a considerable number of false positives, particularly in low prevalence settings.^6^ Published literature for a variety of anti‐HCV tests demonstrates that false positives are more common in “weakly positive” specimens, such as those with low signal cutoff (S/CO) or cutoff index (COI), depending on the methodology employed.^7^, ^8^ Historically, the recombinant immunoblot assay (RIBA) served as a confirmatory step for samples with positive anti‐HCV antibodies; however, the RIBA is no longer routinely available in the United States. Therefore, most laboratories now offer strategies for performing HCV RT‐PCR as confirmatory testing for positive anti‐HCV screening results. There are numerous approaches in use to accomplish this, many of which add complexity and additional expenses to specimen requirements (e.g., collection of a separate specimen tube and freezing of specimens). An alternative approach using a single‐tube reflex to RT‐PCR has been validated and utilized by some institutions to minimize workflow complexity and mitigate the need for additional appointments and sample collections.^9^, ^10^ Additionally, in a population with low HCV prevalence, such reflex testing may result in diversion of resources and increased manual effort and cost, despite the high specificity of contemporary anti‐HCV serological assays.^11^

An alternative screening approach, which has been adopted by several institutions including our own, is to reflexively perform a second anti‐HCV assay for samples with weak positivity on the initial anti‐HCV assay to mitigate false‐positive anti‐HCV results, prior to sending for confirmatory testing by RT‐PCR.^8^, ^12^, ^13^, ^14^ This approach can use the single specimen collected for the initial anti‐HCV screening, without the more stringent specimen requirements needed for RT‐PCR. An additional benefit is that the use of two automated assays can provide results with rapid turnaround time. Here, we report our experience using this two‐step serological screening approach and evaluate its performance in a low prevalence population.

## 2 MATERIALS AND METHODS

### 2.1 Study design

**FIGURE 1:**
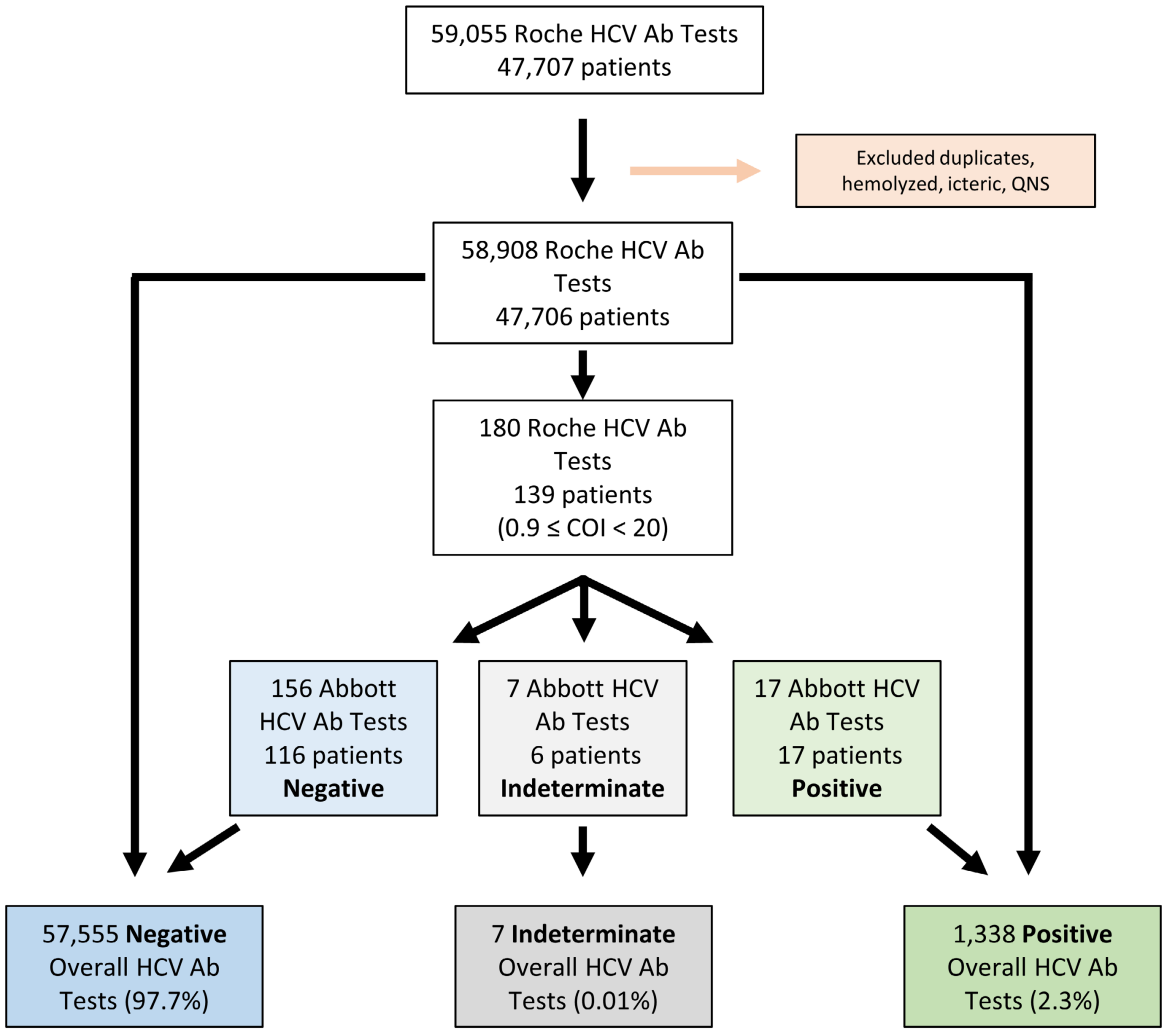
Analysis workflow. A graphical depiction of the data that were collected and analyzed for this study. Bolded terms indicate the results reported to our electronic medical record.

This is a retrospective study performed at an 860 bed tertiary/quaternary care academic medical center in a mostly rural state. The medical center serves as the major referral and specialty hospital in the geographic region. This study was approved by the University of Iowa Institutional Review Board (protocol #202008415) with waiver of informed consent. We queried our institutional EMR database for patients that had HCV screening performed between February 1, 2017, and February 4, 2022, and identified 59,055 samples from 47,707 individual patients. We removed duplicate samples, as well as those that were hemolyzed, icteric, or quantity not sufficient, resulting in a final dataset of 58,908 samples from 47,706 patients (Figure 1). The subset of samples (180 samples from 139 unique patients) that were borderline/weakly positive on initial screening using the Roche assay (see testing methodology) were isolated for chart review including prior HCV testing and history of HCV infection. To evaluate the performance of our diagnostic algorithm, we grouped patients into one of the three groups according to their history of HCV using an approach similar to that employed by others.^15^ Patients were determined to have a history of HCV if they had more than one consecutive positive serological test (any methodology), were found to be HCV positive by RT‐PCR at any point, or had a clearly documented history of HCV in the medical record (multiple unique references). Negative HCV history was defined as uniformly negative serological testing, a lack of documented history (no references by patient or provider), or a single positive serological result with subsequent negative serological testing. Cases that did not fit clearly into either category were considered to have a possible HCV history and given the lack of confidence in their HCV status, were excluded from analyses relying on knowledge of HCV history (e.g., positive predictive value).

### 2.2 Methods

**FIGURE 2:**
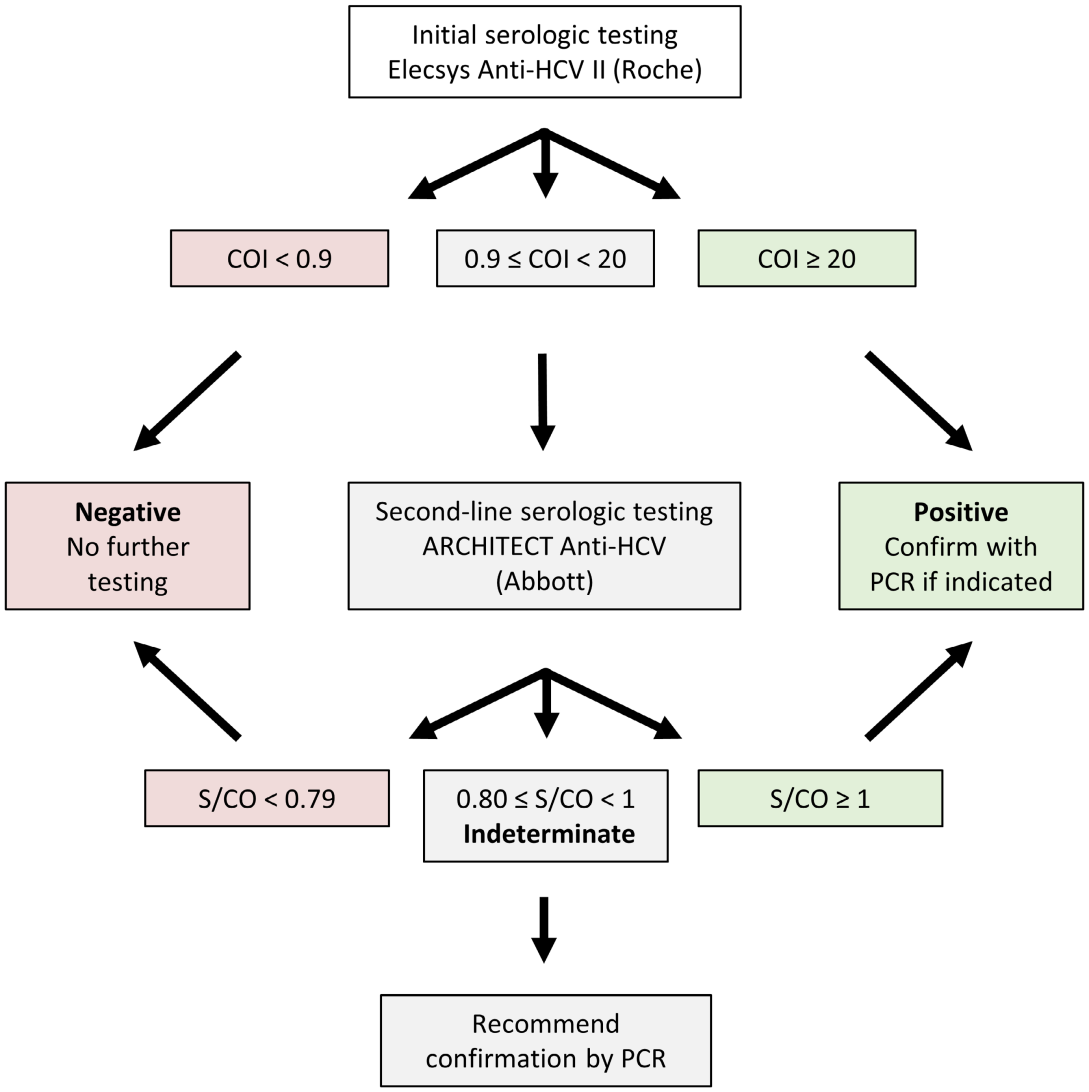
Testing Algorithm. A graphical depiction of the HCV testing algorithm utilized by our institution. Bolded terms indicate the results reported to our electronic medical record based on the interpretation of our two‐assay algorithm.

We adopted a two‐assay testing algorithm, like those described in other studies,^8^, ^12^ in which a second anti‐HCV serological assay is performed on specimens with borderline or weakly positive values on the initial anti‐HCV assay (Figure 2). Patient heparinized plasma are initially analyzed using the Elecsys Anti‐HCV II assay (Roche Diagnostics Elecsys Anti‐HCV II package insert, version 4.0) run on Cobas e602 or e801 automated immunoassay analyzers (Roche Diagnostics). While the sensitivity and specificity of the Roche Anti‐HCV II assay are not reported by the manufacturer in the assay package insert, these test characteristics have been evaluated independently in a separate publication that determined a sensitivity of 99.3% and specificity of 99.86% in a population where the prevalence of HCV was 1%.^15^ The Roche results are reported in the form of a COI, which is the signal detected in the sample divided by the cutoff measured by the instrument based on signal in the negative and positive calibrators. According to the manufacturer's guidelines, COI < 0.9 indicates a negative test, COI ≥ 1 is positive, and values of 0.9 ≤ COI < 1 are considered borderline.

In our two‐assay algorithm, COI values <0.9 are reported to the electronic medical record (EMR, Epic, Inc., version May 2021) as negative and COI values ≥20 are reported as positive; no additional reflexive serological testing is performed in these instances. In contrast, specimens with Roche COI values ranging from 0.9 to 19.99 (i.e., borderline/weakly positive) have previously been shown in other publications to contain many false positives when HCV prevalence is low.^12^ We thus perform reflex testing of specimens with weakly positive anti‐HCV screens by the Roche method with the Abbott ARCHITECT Anti‐HCV assay (ARCHITECT Anti‐HCV assay package insert, 2012) run on an Architect i1000 analyzer (Abbott). In this subset of samples, Abbott anti‐HCV results (reported as S/CO ratio) then dictate the final interpretation in accordance with manufacturer guidelines: a S/CO ratio <0.8 is considered negative, S/CO ≥1 is considered positive, and S/CO ranging from 0.80 to 0.99 is considered indeterminate. Ultimately, indeterminate and positive anti‐HCV values are resulted to the EMR with a comment recommending further testing for HCV RNA by RT‐PCR if clinically indicated. Both Roche and Abbott assays utilize a sandwich immunoassay format to detect antibodies to antigens in the core, N3, and N4 coding regions of HCV.

When performed, HCV RNA is quantified using the COBAS AmpliPrep/COBAS TaqMan HCV test, v2.0. Sensitivity and specificity of this assay are reported to be ~100% with a limit of detection of 15 IU/mL (package insert version 8, 2021).

### 2.3 Statistical analysis

Statistical analyses were performed in GraphPad Prism (version 9.3.1) using nonparametric methods, either the Kruskal–Wallis test for comparison of three or more groups or the Mann–Whitney test for comparison of two groups.

## 3 RESULTS

Our institution utilizes a two‐assay testing algorithm for detecting the presence of anti‐HCV antibodies in patient plasma (Figure 2). We first reviewed the efficacy of this approach by assessing the results of 58,908 anti‐HCV tests from 47,706 patients performed over a 5‐year span. A total of 97.7% (57,555 samples) of anti‐HCV tests were negative (Roche COI < 0.9) and 2.3% (1338 samples) were positive (Roche COI ≥ 20). A total of 0.3% (180 samples) had borderline/weakly positive Roche COI values ranging from 0.9 to 19.99 and were thus tested by the second anti‐HCV assay (Abbott) in our two‐assay algorithm (Figure 1). The baseline characteristics of the patients whose samples are represented in this study (i.e., borderline/weakly positive Roche anti‐HCV) are included in Table 1.

**TABLE 1:** Patient characteristics for samples with borderline/weakly positive Roche anti‐HCV (0.9 ≤ COI < 20).

	Negative	Indeterminate	Positive
Number	156	7	17
Sex %—M/F	54/46	57/43	53/47
Age (range)	52 (0–86)	51 (31–66)	52 (31–74)
HCV history	8	1	11
No HCV history	143	6	6
Possible HCV history	5	0	0

**FIGURE 3:**
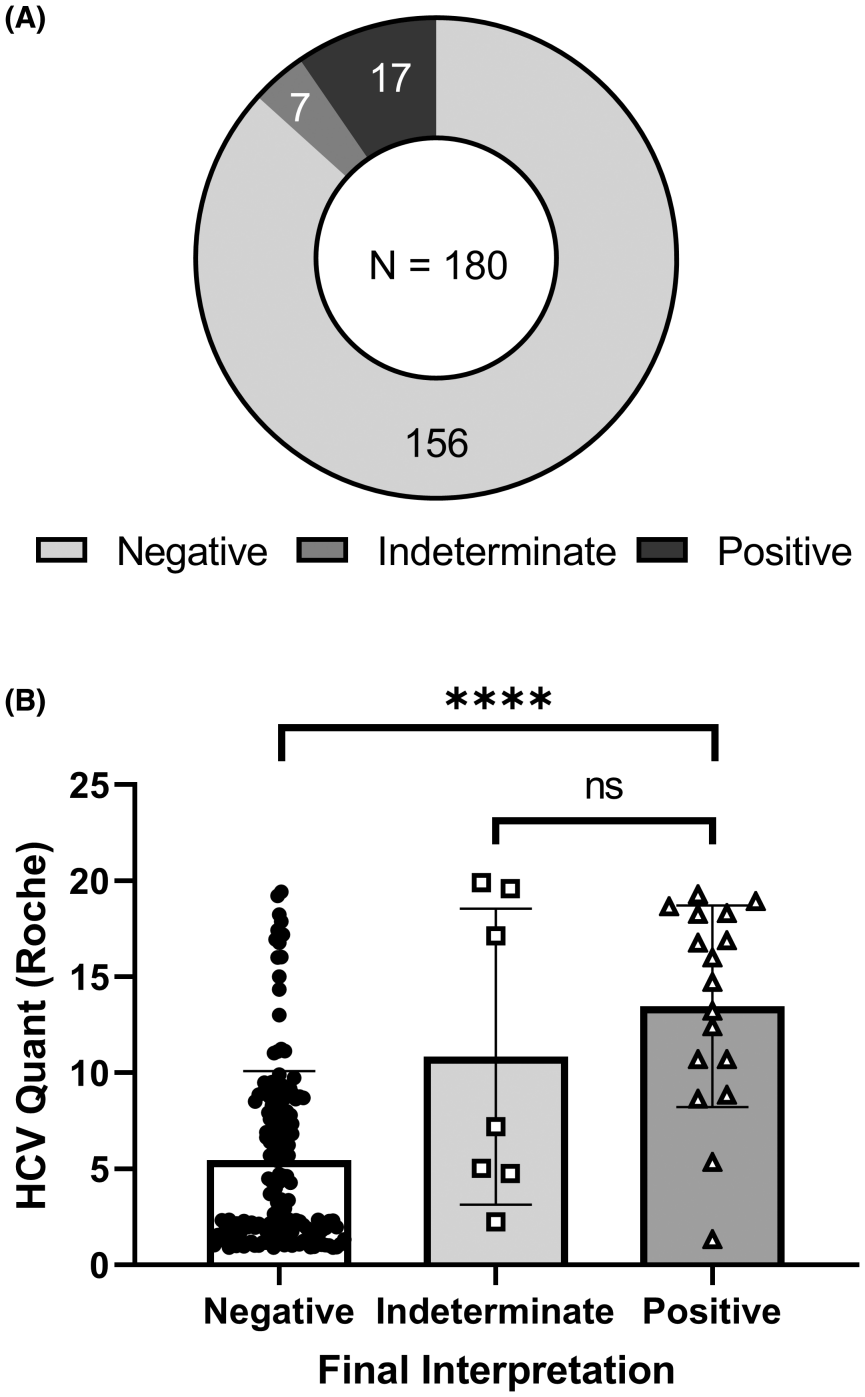
Outcomes of sample with borderline/weakly positive Roche anti‐HCV (0.9 ≤ COI < 20). (A) Final interpretation of samples requiring second‐line serological testing. (B) Results from Elecsys Anti‐HCV II assay for all samples requiring second‐line serological testing grouped by final interpretation. Data are graphed as COI with each result displayed as a single point, and error bars representing standard deviation. Statistics were performed using Kruskal–Wallis test, with **** indicating a *p*‐value <0.0001 and “ns” indicating non‐significance relative to *p*‐value <0.05.

The low prevalence of HCV infection in our patient population (~0.026%, Iowa Department of Public Health, 2020) led us to ask whether our two‐step testing algorithm was beneficial. To address this, we closely examined the subset of samples that required both Roche and Abbott serological assays. These consisted of 180 samples (0.3%) from 139 unique patients that had borderline/weakly positive results using the Roche anti‐HCV assay (0.9 ≤ COI < 20) and were then tested reflexively by the Abbott anti‐HCV assay. From this 180 sample subset, 17 samples (9.4%) with Roche COI ranging from 1.32 to 19.29, all from unique patients, were positive on the Abbott assay (S/CO ≥1), and 156 samples (87%) from 116 patients were negative using the Abbott assay (S/CO < 0.8). Only seven samples (3.9%) were indeterminate on the Abbott assay (0.8 ≤ S/CO < 1) and thus were resulted in the EMR as indeterminate, with additional RT‐PCR testing recommended in a comment (Figure 3A).

Next, we evaluated whether there were quantitative differences in the results of the initial Roche anti‐HCV testing that could be used to predict the final anti‐HCV interpretation. While significantly higher COI values were observed in the group that ultimately resulted as positive by the Abbott assay (mean COI of 13.4 in positives vs 5.5 in negatives; *p* value < 0.00001, Kruskal–Wallis test), there was also notable overlap in the COI distributions (Figure 3B). We noted that several patients had multiple borderline/weakly positive test results on our primary serological assay (Roche), the vast majority of which were ultimately negative by second‐line Abbott testing in our two‐assay algorithm (Table 2). To address whether these samples were biasing our results, we repeated the analysis using the 139 samples remaining after repeat result removal but found similar results (data not shown).

**TABLE 2:** Patient characteristics for those with multiple samples with borderline/weakly positive Roche anti‐HCV (0.9 ≤ COI < 20).

	Sample finalized Results	Testing period (days)	Relevant history	HCV history
Pt. #1	5 negative, 1 indeterminate	597	Fabry disease, ESRD	Yes
Pt. #2	2 negative	545	Esophageal cancer	No
Pt. #3	2 negative	332	None	Possible
Pt. #4	3 negative	236	High risk	No
Pt. #5	1 negative, 1 indeterminate	7	Pregnancy	No
Pt. #6	16 negative	1531	ESRD on dialysis	No
Pt. #7	12 negative	1645	ESRD on dialysis	No
Pt. #8	4 negative	1461	High risk	No
Pt. #9	2 negative	72	High risk	No
Pt. #10	1 negative, 1 indeterminate	7	Liver disease	No

**FIGURE 4:**
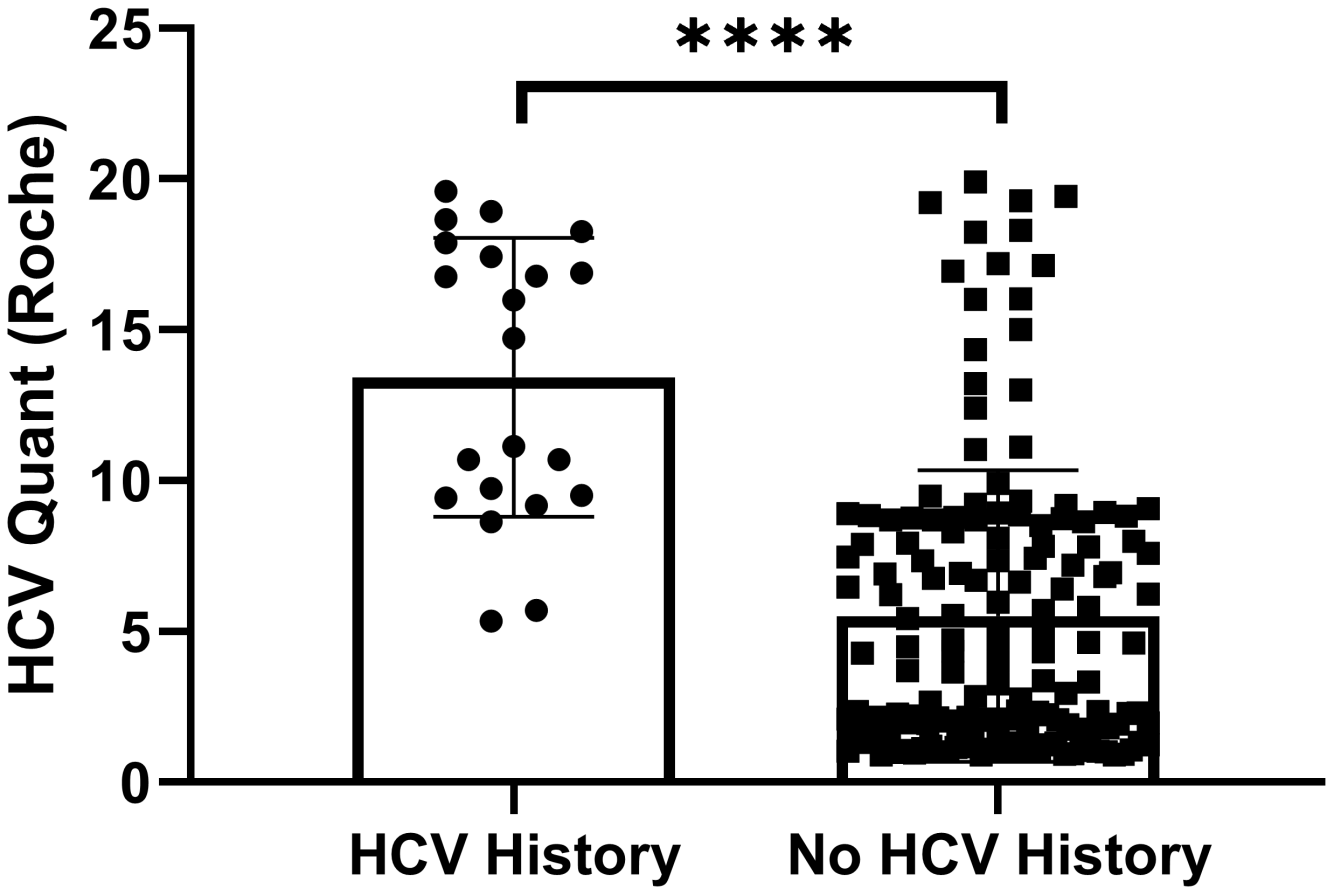
Anti‐HCV screening results grouped by HCV history. Results from Elecsys Anti‐HCV II assay for all samples requiring second‐line testing grouped by history of HCV infection. Data are graphed as COI with each result displayed as a single point, and error bars representing standard deviation. Statistics were performed using Mann–Whitney test, with **** indicating a *p*‐value <0.0001.

We next assessed the concordance between final anti‐HCV interpretation and HCV history based on chart review, as previously defined. Of the 17 samples ultimately interpreted as positive for anti‐HCV, 11 samples (65%), all from unique patients, were found to have a history of HCV. In contrast, only one of seven (14%) indeterminate and eight of 156 (5.1%) negative samples, five of which represented the same patient, had a documented history of HCV, although none of these samples were positive by RT‐PCR at any point. We also compared the quantitative values of the initial Roche testing for samples from patients with and without a history of HCV and found that the Roche COI was significantly higher in the group with a positive history of HCV (median COI of 14.7 in positive HCV history vs 3.7 in no HCV history; *p* value < 0.00001, Mann–Whitney test; Figure 4). Given that confirmation by RT‐PCR is the gold standard for diagnosing HCV infection, we next analyzed the subset of samples from patients that had RT‐PCR performed (*n* = 51). Within this subset, 8/16 (50%) samples ultimately interpreted as anti‐HCV positive were positive by RT‐PCR. In comparison, 0/5 (0%) anti‐HCV indeterminate and 0/30 (0%) anti‐HCV‐negative samples were positive by RT‐PCR.

Finally, as the primary goal of our current algorithm is to enhance the specificity of HCV screening in samples with borderline or weakly positive anti‐HCV results, we sought to directly determine the ability of our two‐assay approach (Roche + Abbott) to predict positive HCV history relative to a single‐assay approach using Roche alone (positive results defined by the Roche recommend cutoff of COI ≥ 1). Our two‐assay approach significantly improved PPV from 12% using Roche only to 65% using Roche and Abbott. When restricting this analysis to the subset of samples from patients with confirmatory RT‐PCR, our two‐assay approach again outperformed Roche alone, improving PPV from 16% to 50%.

## 4 DISCUSSION

Acute HCV infection often causes a mild or moderate self‐limiting illness with nonspecific symptoms that can be easily missed. While many acute infections are cleared spontaneously, over 50% of infections persist in the form of chronic hepatitis, which is frequently asymptomatic until irreversible complications develop.^16^ Effective screening programs are therefore vital to facilitating diagnosis and treatment. Here, we explored the benefit of a two‐step serological screening algorithm for weakly positive anti‐HCV samples in a population with low HCV prevalence (<0.05%).

In our study, the major difference between a single‐assay and two‐assay approach is in the number of false positives. If we had followed the Roche assay package insert for COI interpretation, eight samples would have been resulted as “borderline” (0.9 ≤ COI < 1), compared to seven samples that underwent two‐assay testing and were eventually found to be indeterminate by the Abbott method. In contrast, if we had used the Roche assay alone, 172 of the 180 samples in our dataset would have been reported as positive according to manufacturer's instructions, with only 20 being true positives (PPV = 12%) based on chart review of additional clinical and laboratory information. In comparison, 11 of 17 samples tested by both assays that were positive by the Abbott assay were truly positive (PPV = 65%) based on chart review. Even when we restrict our analysis to samples from patients with confirmatory RT‐PCR, and therefore the cases with the highest degree of confidence in true HCV infection, the PPV of Roche alone (8/51, PPV = 16%) is significantly lower than the PPV using our two‐assay approach (8/16, PPV = 50%). This is critical as false‐positive tests can lead to misdiagnoses inflicting significant physical and emotional burden to the patient until further diagnostic workup can be performed. Another advantage of our approach is that both tests can be performed on the same sample, an important consideration as the populations at the highest risk for HCV infection are more likely to have difficulty accessing health‐care services and may be more easily lost to follow‐up. While it is true that reflexively testing by a secondary anti‐HCV assay for samples with Roche COI 0.9≤ to <20 imposes some burden on laboratory staff in the form of extra testing and delays overall anti‐HCV reporting turnaround time, this affected only 180 samples over a 5‐year period in our population. In addition, the instrumentation used for the second serologic assay was already used by our laboratory for other testing, a key consideration for others considering implementation of such an approach.

Our study has limitations meriting discussion. First, this study was performed at a single center in Iowa, and thus the number of samples are smaller and from a less diverse population than could be obtained in a multicenter study. Another limitation is that it is functionally impossible to be certain as to the true HCV status of most patients undergoing HCV screening, especially as current guidelines suggest no further testing following negative serology. A similar approach to determine HCV status to the one used here has been employed previously and served as a model for our study.^15^ It is certainly encouraging that patients with a presumed history of HCV have statistically higher COI by the Roche assay than those without, but false negatives are still possible with our approach (Figure 4). Third, our borderline/weakly positive Roche COI range warranting additional anti‐HCV testing by a secondary assay (i.e., 0.9 ≤ COI < 20) may not be optimal. Based on our analyses described herein, there are likely false‐positive Roche anti‐HCV results with COI ≥ 20 that would also benefit from reflexive testing by the Abbott anti‐HCV assay. Finally, we can only compare our two‐assay approach to a Roche‐only approach within the confines of our 180 sample subset as we do not have Abbott results for the remaining samples that were either definitively negative (COI < 0.9) or more strongly positive (COI ≥20) on the Roche anti‐HCV assay. Similarly, we cannot definitively calculate sensitivity and specificity or generate a ROC curve for this approach as our algorithm dictates that a negative anti‐HCV screening assay warrants no further confirmatory testing. Thus, we cannot identify false negatives, precluding these calculations. While it would be of interest to compare these methods directly, the logistics of obtaining the required data on ~60,000 samples, either by chart review or additional testing, preclude such an endeavor. It is worth noting that others have taken a similar approach to first‐line testing and have found the sensitivity and specificity of the Roche Anti‐HCV II assay to be 99.3% and 99.86%, respectively, in a population with a high prevalence of HCV infection.^14^ Using our two‐assay approach, we would predict that specificity would be enhanced, a critical advantage when testing in low prevalence areas.

Taken together, we demonstrate that a two‐assay serological testing algorithm for weakly positive anti‐HCV samples significantly increases the PPV of HCV screening when compared to a single‐assay approach using manufacturer's guidelines. We find that the extra testing required by such an approach comes at a minimal cost in our low prevalence population, affecting only 180 samples in 5 years. By reducing the number of false positives in specimens with borderline or weak anti‐HCV positivity, this approach significantly reduces unnecessary follow‐up requiring additional health‐care services and particularly improves the quality of care provided to vulnerable patients at highest risk for HCV infection.

## CONFLICT OF INTEREST STATEMENT

None declared.

## Data Availability

The data that support the findings of this study are available from the corresponding author upon reasonable request.
